# MiR-133b Targets Antiapoptotic Genes and Enhances Death Receptor-Induced Apoptosis

**DOI:** 10.1371/journal.pone.0035345

**Published:** 2012-04-20

**Authors:** Juan P. Patron, Annika Fendler, Matthias Bild, Ulrike Jung, Henrik Müller, Magnus Ø. Arntzen, Chloe Piso, Carsten Stephan, Bernd Thiede, Hans-Joachim Mollenkopf, Klaus Jung, Stefan H. E. Kaufmann, Jörg Schreiber

**Affiliations:** 1 Department of Immunology, Max Planck Institute for Infection Biology, Berlin, Germany; 2 Department of Urology, Charité, University Medicine, Berlin, Germany; 3 Berlin Institute for Urologic Research, Charitéplatz 1, Berlin, Germany; 4 Department of Biology, Chemistry and Pharmacy, Free University, Berlin, Germany; 5 The Biotechnology Centre of Oslo, University of Oslo, Oslo, Norway; 6 Proteomics Core Facility, Oslo University Hospital-Rikshospitalet and University of Oslo, Oslo, Norway; 7 Proteomics Core Facility, Norwegian University of Life Sciences, Aas, Norway; 8 Core Facility Microarray, Max Planck Institute for Infection Biology, Berlin, Germany; University of Hong Kong, Hong Kong

## Abstract

Despite the importance of microRNAs (miRs) for regulation of the delicate balance between cell proliferation and death, evidence for their specific involvement during death receptor (DR)-mediated apoptosis is scarce. Transfection with miR-133b rendered resistant HeLa cells sensitive to tumor necrosis factor-alpha (TNFα)-induced cell death. Similarly, miR-133b caused exacerbated proapoptotic responses to TNF-related apoptosis-inducing ligand (TRAIL) or an activating antibody to Fas/CD95. Comprehensive analysis, encompassing global RNA or protein expression profiling performed by microarray experiments and pulsed stable isotope labeling with amino acids in cell culture (pSILAC), led to the discovery of the antiapoptotic protein Fas apoptosis inhibitory molecule (FAIM) as immediate miR-133b target. Moreover, miR-133b impaired the expression of the detoxifying protein glutathione-*S*-transferase pi (GSTP1). Expression of miR-133b in tumor specimens of prostate cancer patients was significantly downregulated in 75% of the cases, when compared with matched healthy tissue. Furthermore, introduction of synthetic miR-133b into an *ex-vivo* model of prostate cancer resulted in impaired proliferation and cellular metabolic activity. PC3 cells were also sensitized to apoptotic stimuli after transfection with miR-133b similar to HeLa cells. These data reveal the ability of a single miR to influence major apoptosis pathways, suggesting an essential role for this molecule during cellular transformation, tumorigenesis and tissue homeostasis.

## Introduction

Apoptosis, a strictly regulated form of active programmed cell death (PCD), plays crucial roles in a plethora of both homeostatic and pathological processes in multicellular organisms [1]. Apoptotic cells are characterized by well-defined morphological changes some of which include rounding-up of the cell, reduction of cellular volume, chromatin condensation, nuclear fragmentation and membrane blebbing [2]. This process of controlled cellular suicide can be triggered by extracellular and intracellular stimuli, both of which result in activation of specific, yet partially overlapping signaling cascades. Death receptors (DRs) represent a group of extracellular membrane-bound molecules responsible for sensing and transducing exogenously derived proapoptotic signals. DRs, including tumor necrosis factor receptor 1 and 2 (TNFR1/2), Fas/CD95 and TNF-related apoptosis-inducing ligand (TRAIL) receptors DR4 and DR5, belong to the TNF superfamily and share a common structurally conserved 80 amino acid-long cytoplasmic death domain (DD) [3]. Upon cognate ligand binding, DRs oligomerize via their DD giving rise to a scaffold for the recruitment of several adaptor and signaling molecules. At this death-inducing signaling complex (DISC), initiator caspases such as caspase 8 and 10 become activated by autocatalytic cleavage. Once triggered, initiator caspases initiate the execution phase of the death signal by processing effector caspases, like caspase 3, 7 or 9, to their active forms. As a consequence of this released proteolytic activity, caspase substrates including key intracellular factors are degraded and the cell is inevitably committed to death [4]. Cells harbor a complex set of mechanisms aimed at regulating their responsiveness to DR ligands. For instance, cells can avoid initiation of the proapoptotic program by survival factors that impair caspase activation. The relevance of apoptosis and the proper function of its negative regulators for systemic homeostasis are exemplified by human patients suffering from devastating diseases like cancer, neurodegeneration or autoimmunity.

MicroRNAs (miRs) are endogenous short (∼22 nt) RNA molecules that play an essential role in regulation of cellular processes [5]. To date, the best characterized function of miRs is fine tuning of gene activity at the post-transcriptional level. To this end mature miRs are incorporated into an elaborate ribonucleoprotein structure termed RNA-induced signaling complex (RISC). Once RISC is loaded with an miR, it exploits its ‘seed sequence’ to find matching mRNAs. Depending on the degree of complementarity between the miR and its target, mRNA expression is blocked either through direct cleavage or translational arrest [6]. Although several miRs are capable of controlling pro- or antiapoptotic processes, the role of miRs in regulation of DR-triggered apoptosis remains elusive [7].

MiR-133b and -206 comprise a bi-cistronic miR cluster originally suggested to be solely expressed in skeletal muscle [8]. Current studies support a broader expression pattern of this cluster and attribute miR-206 important regulatory functions in tissues as diverse as brain, skeletal muscle or adipose tissue [8], [9], [10]. Moreover, miR-206 activates apoptosis and inhibits tumor cell migration and focus formation [11]. MiR-133b, the other cluster's member, is expressed in T-cells [12] and is downregulated during head and neck/oral, bladder, human non-small cell lung, colorectal and esophageal squamous cell cancer [13], [14], [15], [16], [17], [18], [19], [20]. MiR-133b targets important sentinels of mitochondrial membrane integrity such as induced myeloid leukemia cell differentiation protein (MCL-1) and BCL2-like 2 (BCL2L2) and the oncogenes Fascin homolog 1 (FSCN-1) and tyrosine protein kinase c-Met (c-MET) [17], [19], [21]. More recently, and diverging from the aforementioned findings a protumorigenic role of miR-133b was found in cervical cancer [22]. Herein, we characterized miR-133b in the context of DR-mediated apoptosis and prostate cancer. We provide conclusive mechanistic evidence for miR-133b as a regulator of proapoptotic signaling events that apparently play an important role during cancerogenesis of the human prostate.

## Results

### MiR-133b sensitizes cells to DR-mediated apoptosis

**Figure 1:**
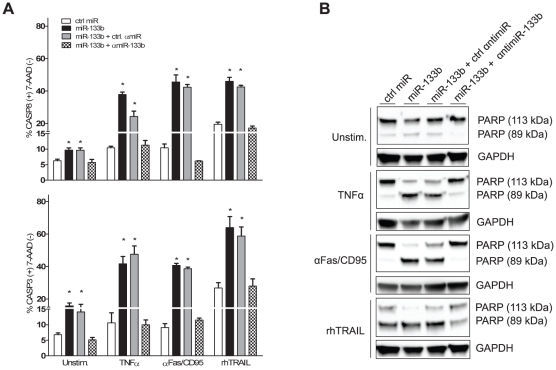
miR-133b acts proapoptotically on HeLa cells stimulated with death receptor (DR) ligands. Cells were transfected with miR-133b alone or together with a control antimiR (ctrl αmiR) or a specific miR-133b inhibitor (αmiR-133b). After 48 h, cells were either left untreated (Unstim) or stimulated for 4 h with 20 ng/ml tumor necrosis factor-alpha (TNFα), 100 ng/ml of a cross-linking activating antiFas antibody (αFas/CD95) or 20 ng/ml recombinant human TRAIL (rhTRAIL). (A) Treated cells were harvested, stained and scanned by flow cytometry for the presence of cleaved active caspase 8 (upper graph) and 3 (lower graph). 7-Amino-actinomycin D (7-AAD) served for exclusion of cells with compromised membrane integrity from the caspase activation quantification assay. Cells transfected with ctrl miR alone were used as reference. (B) Western blot analysis of poly [ADP ribose] polymerase (PARP-1) in transfected, unstimulated cells (upper panel) and TNFα-, αFas/CD95- or rhTRAIL-treated cells (lower panel). Glyceraldehyde 3-phosphate dehydrogenase (GAPDH) was used as an internal loading control. Graphs are representative of at least three independent experiments. Asterisk represents p<0.01. Errors bars indicate standard deviation.

In order to assess whether miR-133b possesses proapoptotic properties, we transfected HeLa cells with a synthetic miR-133b mimic or a negative scrambled control (ctrl miR), stimulated them with TNFα and characterized the cellular response by measuring independent apoptosis markers. In HeLa cells, TNFα-induced apoptosis can be blocked in a NF-κB-dependent manner [23]. Upon activation, NF-κB is released from its inhibitor, translocates to the nucleus and induces expression of antiapoptotic molecules [24]. After transfection with miR-133b, this antiapoptotic response could be bypassed, rendering cells sensitive to TNFα-triggered caspase 8 and 3 activation (Figure 1A). In line with this, poly [ADP ribose] polymerase 1 (PARP-1) cleavage, a hallmark of apoptotic cells [25], could only be detected in miR-133b transfectants (Figure 1B). Both effects took place in a sequence-specific manner, since transfection of ctrl miR did not result in altered activation status of initiator and executer caspases or PARP-1 degradation. Moreover, TNFα sensitization could be inhibited by adding a specific miR-133b inhibitor (αmiR-133b), but not a random control sequence (ctrl αmiR) [26], [27]. Remarkably, activation status of caspase 8 and 3 in unstimulated cells, as well as the amount of cleaved PARP-1, were also significantly and specifically higher only after miR-133b transfection. This effect could be blocked in a sequence-specific manner by introduction of αmiR-133b (Figure 1A and B). We next inquired whether miR-133b could also affect cellular responses to other DR ligand family members. Comparable to TNFα resistance, Fas ligand (FasL) refractory cells do not undergo apoptosis upon receptor ligation [28]. MiR-133b transfection reversed this phenotype and induced a 5-fold stronger activation of caspase 8 and 3, together with PARP-1 depletion, after treatment of cells with a cross-linking antiFas/CD95 (αFas/CD95) antibody. TRAIL-stimulated cells exhibited a basal level of caspase activation and PARP cleavage, which was potentiated following introduction of miR-133b. In both cases, effects were sequence-specific and could be fully reversed by cotransfection of fully complementary αmiR, but not by a negative control (Figure 1A and B).

**Figure 2:**
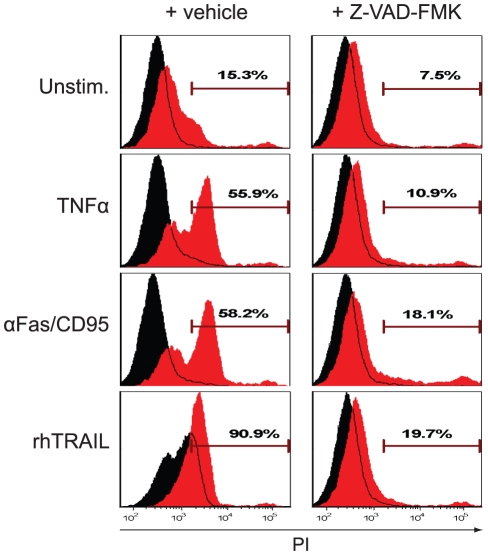
Transfection of HeLa cells with miR-133b results in enhanced caspase-dependent loss of membrane integrity after DR ligation. Cells were transfected with miR-133b alone or together with ctrl miR. After 48 h, cells were left untreated (Unstim) or stimulated for 4 h with either 20 ng/ml TNFα, 100 ng/ml αFas/CD95 or 20 ng/ml rhTRAIL. In order to assess caspase dependency, 0.01% DMSO (vehicle) (left histogram column) or Z-VAD-FMK (50 µM) (right histogram column) were added to the cells. Following stimulation, both adherent and suspension cells were collected, stained with propidium iodide (PI) and analyzed by flow cytometry. Samples were compared to equally treated ctrl miR-transfected cells (black histogram). Results are representative of at least three independent experiments.

Late apoptotic cells are characterized by compromised plasma membrane integrity [29]. To test whether miR-133b insertion leads to promiscuous rupture of the cellular envelope, transfected cells were stimulated with different DR ligands and stained with propidium iodide (PI). Whereas ctrl miR-treated cells hardly stained positive for PI after TNFα or αFas/CD95 treatment, miR-133b led to a marked increase of the PI-positive population under the same conditions. Loss of plasma membrane integrity was also much stronger in TRAIL-treated miR-133b-transfected cells (Figure 2). Importantly, and verifying the proapoptotic nature of miR-133b, pre-treatment with a cell permeable nonselective caspase inhibitor (Z-VAD-FMK) almost completely rescued cellular resistance to DR stimulation (Figure 2).

### Fas apoptosis inhibitory molecule (FAIM) is directly regulated by miR-133b

Next, we questioned which genes are directly targeted by miR-133b. Whole genome microarray expression analysis allowed us to record mRNAs with impaired expression after miR-133b transfection (Microarray data have been deposited in GEO GSE24613. The data are MIAMI compliant and are available through the following link: http://www.ncbi.nlm.nih.gov/geo/query/acc.cgi?token=jnadregqswauixm&acc=GSE24613). Assuming that miR-133b primarily acts by restraining induction of canonical antiapoptotic factors, cells were stimulated with TNFα for 6 h prior to RNA collection. Under these conditions a total of 305 genes (for a detailed gene list please refer to deposited microarray files) emerged as downregulated (FCH<−1.5; p-value<0.001). We also obtained 409 induced genes, but as miRNAs are, in general, supposed to repress gene expression, we focused on downregulated genes in our further analysis. Consistent with published results, the observed mRNA changes were not drastic and peaked at a minimum of −4.8 fold [30]. In order to filter the data for genes with the necessary sequence features to be considered as potential miR-133b targets, we matched the list of downregulated genes with miRecords, an miR target prediction database (Table 1). This online accessible repository is an archive of results produced by 11 established miR target prediction programs [31]. Given the proapoptotic nature of miR-133b, the antiapoptotic gene Fas apoptosis inhibitory molecule (FAIM) captured our attention as an interesting miR-target candidate.

**Table 1:** Genes downregulated after transfection of HeLa cells with miR-133b and TNFα stimulation and predicted to be potential miR targets.^1^

Symbol	MiRanda	MiRtarget2	Pita	Target scan	FCH array	p-value
ACAT2	YES	YES	YES		−1.6	8E-08
MYH9	YES		YES	YES	−1.6	7E-07
SLC30A7	YES	YES	YES		−1.6	3E-09
CPNE3	YES	YES	YES		−1.7	3E-21
ARL13B	YES	YES	YES		−1.7	9E-21
FOXP4	YES		YES	YES	−1.7	6E-08
ARL14	YES	YES	YES		−1.7	8E-09
COQ7	YES	YES	YES		−1.7	9E-13
TOR1AIP2	YES	YES	YES		−1.8	5E-22
EFEMP1	YES	YES	YES		−1.8	1E-24
SLC2A12	YES		YES	YES	−1.9	7E-09
CETN3	YES	YES	YES		−1.9	3E-11
**FAIM**	**YES**	**YES**	**YES**	**YES**	−**2.0**	**3E-20**
CREB5	YES		YES	YES	−2.3	8E-11
CTH	YES	YES	YES		−2.5	2E-33
KLRK1	YES	YES	YES		−3.4	3E-37

1 According to the online database miRecords and predicted at least by three different algorithms.

**Figure 3:**
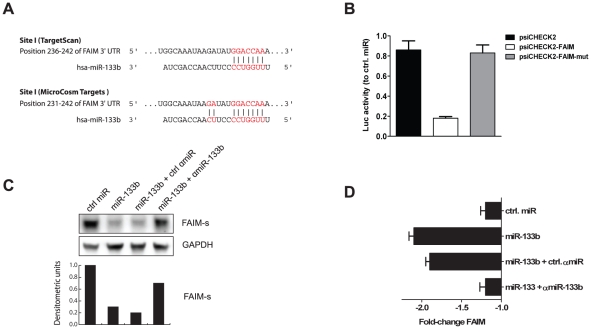
Fas apoptosis inhibitory molecule (FAIM) expression is regulated by miR-133b. (A) miR-133b target sites within the 3′-UTR of FAIM as predicted by TargetScan and MicroCosm Targets. (B) 3′-UTR targeting assay. HeLa cells were cotransfected with different combinations of miR mimics and a luciferase reporter plasmid harboring the complete 3′-UTR of FAIM (psiCHECK2-FAIM) or the mutated version thereof (psiCHECK2-FAIM-mut). Template plasmid (psiCHECK2) was used as a negative and normalization control. Activity of the 3′-UTR-dependent luciferase (*Renilla reniformis*) was measured 48 h post-transfection and normalized for transfection efficiency to the one produced by the miR- and 3′-UTR-independent luciferase (*Photinus pyralis*). Error bars indicate standard deviation. (C) Western blot and densitometric analysis of FAIM expression. Cells were transfected with miR-133b alone or together with ctrl αmiR or specific αmiR-133b. After 48 h, cellular protein lysates were prepared and FAIM expression was assessed by Western blot. GAPDH was used as an internal loading standard. Ctrl miR-transfected cells were used as a reference for densitometric quantification of protein band intensity. (D) qPCR analysis. Cells were transfected for 48 h before total RNA was isolated, reverse transcribed and analyzed by qPCR for expression of FAIM. Human acidic ribosomal protein (HuPO) was used as the housekeeping gene for internal normalization. Fold-change (FCH) values are shown relative to mock-transfected cells incubated under the same conditions. All graphs are representative of at least three independent experiments.

FAIM is a widely expressed and evolutionarily conserved protein originally cloned from B cells and with protective traits against Fas/CD95-mediated apoptosis [32]. The 3′-UTR region of FAIM contains one single miR-133b binding site (Figure 3A). Cloning of complete 3′-UTR of FAIM into psiCHECK-2 luciferase reporter plasmid reduced *Renilla* luciferase activity to 19% after cotransfection of miR-133b. Interaction between the binding-site in the 3′-UTR and miR-133b was sequence-specific, since mutation of the seed sequence restored *Renilla* luciferase activity (Figure 3B) to values comparable to the empty psiCHECK vector. Moreover, as predicted by microarray analysis, miR-133b transfection of HeLa cells translated into specific downregulation of FAIM protein as demonstrated by Western blot analysis (Figure 3C). On the mRNA level quantitative PCR (qPCR) also revealed reduced amounts of FAIM molecules relative to human acidic ribosomal protein (HuPO) as a house keeping gene (Figure 3D).

### MiR-133b impairs expression of glutathione-*S*-transferase pi (GSTP1)

**Figure 4:**
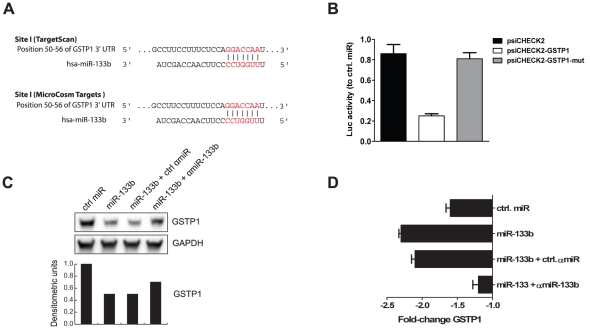
miR-133b controls expression of glutathione-*S*-transferase pi (GSTP1). (A) miR-133b target sites within the 3′-UTR of GSTP1 as predicted by TargetScan and MicroCosm Targets. (B) 3′-UTR targeting assay. HeLa cells were cotransfected with different combinations of miR mimics and a luciferase reporter plasmid harboring the complete 3′-UTR of GSTP1 (psiCHECK2-GSTP1) or the 3′-UTR missing the miR-133b binding site (psiCHECK2-GSTP1-mut). Template plasmid (psiCHECK2) was used as a negative and normalization control. Activity of the 3′-UTR-dependent luciferase (*Renilla reniformis*) was measured 48 h post-transfection and normalized for transfection efficiency to the one produced by the miR- and 3′-UTR-independent luciferase (*Photinus pyralis*). Error bars indicate standard deviation. (C) Western blot and densitometric analysis of GSTP1 expression. Cells were transfected with miR-133b alone or together with ctrl αmiR or a specific αmiR-133b. After 48 h, cellular protein lysates were prepared and GSTP1 expression was assessed by Western blot. GAPDH was used as an internal loading standard. Ctrl miR-transfected cells were used as a reference for densitometric quantification of protein band intensity. (D) qPCR analysis. Cells were transfected for 48 h before total RNA was isolated, reverse transcribed and analyzed by qPCR for the expression of GSTP1. HuPO was used as the housekeeping gene for internal normalization. Fold-change (FCH) values are shown relative to mock-transfected cells incubated under the same conditions. All graphs are representative of at least three independent experiments.

Microarray analysis of miR-mediated gene regulation relies exclusively on mRNA decay. Since miR-mediated gene expression repression also occurs at the translational level, we decided to perform pulsed stable isotope labeling with amino acids in cell culture (pSILAC) analysis of HeLa cells transfected with miR-133b or control miR [33]. As done previously, transfected cells were stimulated with TNFα for 6 h prior to protein lysate harvest. In accordance with previous reports about quantitative effects of miR activity on the cellular proteome, ectopic introduction of miR-133b into HeLa cells led only to mild effects on overall protein biosynthesis [33]. Among the most strongly repressed proteins, glutathione-*S*-transferase pi (GSTP1) constituted an interesting target gene, since it represents an important factor fulfilling protective and detoxifying functions in tumor cells [34]. In line with this, cross-comparison of pSILAC results with microarray expression analysis revealed repression of GSTP1 at the mRNA level (Table 2). This had not been identified previously, because of the filtering strategy applied to the miR-133b target predictions according to miRecords (*i.e.* target prediction by at least three independent tools; see Table 1). According to the *in-silico* prediction tools TargetScan and MicroCosm Targets, GSTP1 displays one single miR-133b binding site in its 3′-UTR (Figure 4A) [35], [36], [37]. As demonstrated by luciferase reporter assays, coupling of entire GSTP1 3′-UTR to *Renilla* luciferase specifically rendered it less active after cotransfection of miR-133b. Supporting a direct interaction between miR-133b and the cloned target sequence, mutation of the predicted seed sequence reinstated enzymatic activity (Figure 4B). Moreover, qPCR analysis corroborated the suppressive effect of miR-133b on the mRNA levels of GSTP1 (Figure 4D). As the final line of evidence, Western blot analysis confirmed the pSILAC results by proving protein levels of GSTP1 to be miR-133b sensitive (Figure 4C) in HeLA cells. Using pSILAC FAIM was not identified.

**Table 2:** Apoptosis regulatory proteins downregulated in miR-133b-transfected TNFα-stimulated HeLa cells.

UniProt name	1Fold repression	Positive correlation with microarray analysis	2Predicted miR-133b targets
TAGLN2	4.2	YES	YES
MYH9	2.7	YES	YES
CKAP4	2.3	YES	YES
PTBP1	2.2	YES	YES
**GSTP1**	**2.1**	YES	YES
CPNE3	1.9	YES	YES

1 Compared to ctrl miR-transfected and TNFα-stimulated cells. P-value<0.01.

2 As predicted by either TargetScan or MicroCosm Targets.

### MiR-133b is downregulated in prostatic tissue from cancer patients and induces proliferation arrest in PC3 cells

**Figure 5:**
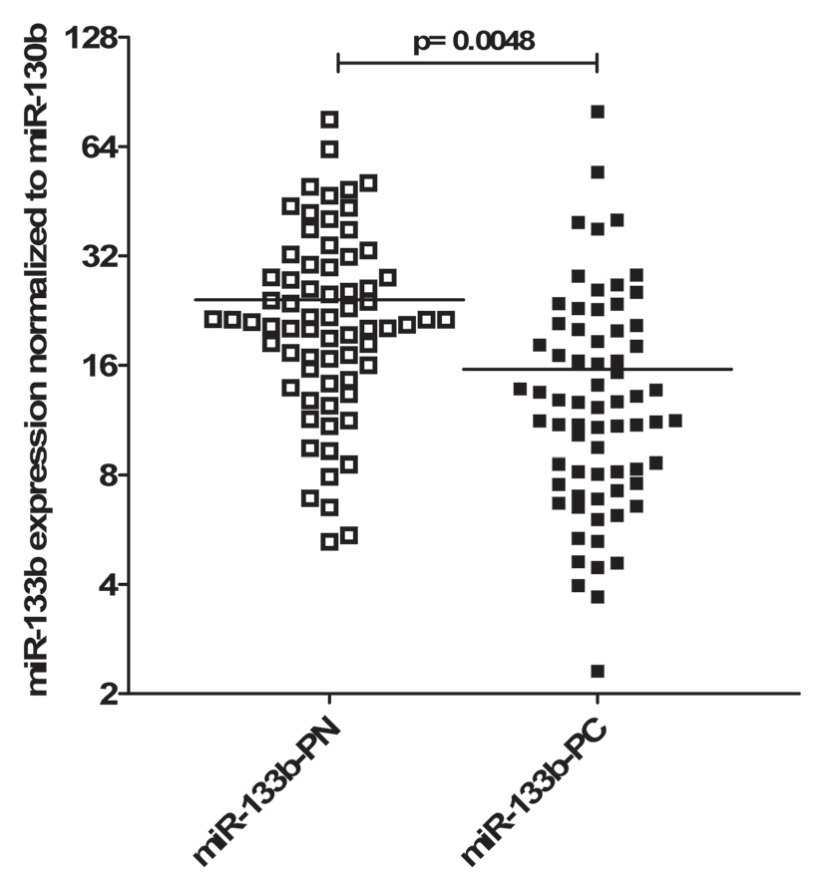
Samples from human prostate cancer patients have decreased expression of miR-133b. Scatter dot-plot of miR-133b expression. RT-qPCR was used to determine miR-133b expression in 69 primary prostate cancer tissues and corresponding normal adjacent tissue. Lines represent mean expression values. MiR-133b was normalized to miR-130b, since it shows a very stable expression pattern both in tumors and normal tissue.

Previous studies described the downregulation of miR-133b in cancer and discussed the potential of such miR signatures for diagnosis and prognosis [13], [14], [15], [16], [17], [18], [19], [21], [38]. To prove the biological significance of miR-133b dysregulation in cancer, we examined miR-133b expression in 69 human prostate cancer samples and matched normal adjacent tissue. Although normalized c_t_-values varied in individual patients, mean miR-133b expression was 1.7-fold decreased in tumor tissue (Wilcoxon signed rank test; p<0.01), with 75% of examined patients displaying lower expression (Figure 5). We did not observe a significant correlation with Gleason Score (r_s_ = −0.20; p = 0.10) or pathological stage (r_s_ = −0.16; p = 0.18). ROC analysis and univariate logistic regression revealed robust discrimination between tumor and normal tissue by miR-133b with an area under the curve (AUC) of 0.73 (p<0.001) and an overall correct classification of 68% (HR = 0.94; 95% CI = 0.91–0.98; p<0.01) (Figure S1). To estimate whether patients with lower miR-133b expression have a higher recurrence incidence, we dichotomized patients according to median expression ratio in tumor tissue (T) versus normal adjacent tissue (N) (Figure S2). Patients with a low T/N ratio had significantly worse recurrence-free survival than patients with high T/N ratio (log rank test; p<0.05). Univariate Cox regression of continuous miR-133b ratio showed borderline significance of miR-133b as predictive marker for biochemical relapse (HR = 0.16; 95% CI = 0.02–1.06; p = 0.06).

**Figure 6:**
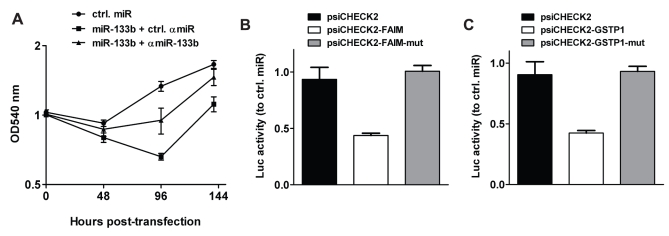
Prostate cancer cells treated with miR-133b show impaired vitality. (A) MTT proliferation assay. PC3 cells were transfected with ctrl miR (•) or, miR-133b together with ctrl αmiR (▪) or with a specific αmiR-133b (▴). Cellular viability and proliferation were determined 0, 48, 96 and 144 h post-transfection as described in Material and Methods. (B) 3′-UTR targeting reporter assay. PC3 cells were transfected with miR mimics and a luciferase reporter plasmid encompassing the complete 3′-UTR of FAIM (psiCHECK2-FAIM) or the 3′-UTR missing the miR-133b binding site (psiCHECK2-FAIM-mut). The empty psiCHECK2 vector was used as a negative control. Error bars indicate standard deviation. (C) Congruent to (B) except that luciferase assays were performed with a construct harboring the GSTP1 3′-UTR.

**Figure 7:**
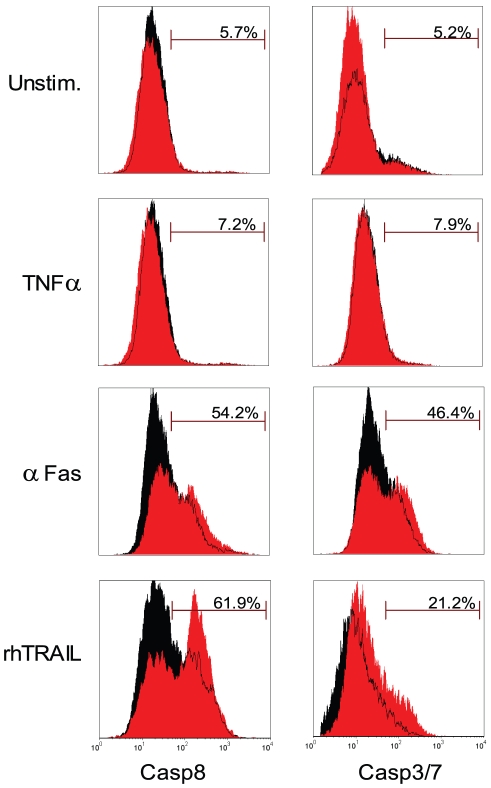
miR-133b induces increased apoptosis in DR receptor stimulated PC3 cells. Cells were transfected with miR-133b (red) or a control miR (ctrl miR, black), as reference. After 48 h, cells were either left untreated (Unstim) or stimulated for 4 h with 20 ng/ml tumor necrosis factor-alpha (TNFα), 100 ng/ml of a cross-linking activating antiFas antibody (αFas/CD95) or 20 ng/ml recombinant human TRAIL (rhTRAIL). Treated cells were harvested, stained and scanned by flow cytometry for the presence of cleaved active caspase 8 (left) and caspase 3 (right). 7-Amino-actinomycin D (7-AAD) was used for exclusion of cells with compromised membrane integrity from the caspase activation quantification assay.

Having proven miR-133b dysregulation in prostate cancer specimens, we addressed the question, whether reduced expression levels of proapoptotic miR-133b could be a mechanism that allows prostate cancer cells to circumvent cell death resulting in higher proliferation rate. Therefore, we monitored *ex-vivo* the metabolic activity of transfected PC3 cells for 6 days. Two days after transfection, minor growth reduction could be observed in all groups, which might have been due to slight cytotoxicity triggered by the transient transfection procedure used. Afterwards, ctrl miR-transfected cells resumed normal proliferation, while growth of cells simultaneously treated with miR-133b and ctrl miR remained repressed. Cotransfection of miR-133b and αmiR-133b partially abolished this inhibition, suggesting a sequence-specific effect (Figure 6A). MiR-133b-mediated growth inhibition was relieved after 96 h most likely due to the transient transfection protocol used in this study. Indeed, also in apoptosis-inducing experiments shown above, the proapoptotic effect catalyzed by miR-133b was strongly reduced or even lost 96 h post-transfection (data not shown). Similarly to the experiments performed in HeLa cells; we then determined whether FAIM and GSTP1 represent miR-133b target genes in prostate cancer cells. To this end, we transfected the wildtype and binding site mutated reporter constructs in PC3 cells. As depicted in Figure 6B and C, luciferase activity was only reduced if reporter vectors harboring the wildtype 3′-UTR were applied. This regulation was not only confined to the reporter constructs but extends to the native proteins as revealed by Western blots with GSTP1- or FAIM-specific antibodies (Figure S3 and Figure S4). Next we examined if miR-133b is also involved in DR-mediated apoptosis in prostate cancer cells. PC3 cells were treated with DR ligands and active caspase levels were determined (Figure 7). Following treatment with a combination of miR-133b and TRAIL or αFas/CD95 antibodies, cellular apoptosis increased as compared to ctrl miR, although this effect was less pronounced than in HeLa cells. In contrast to HeLa cells, stimulation with TNFα did not result in substantial apoptosis under the experimental conditions. This could be attributed to the chosen TNFα concentration and incubation times, which were optimized for HeLa cells and lower than the ones used for PC3 cells in other reports [39]. We conclude that miR-133b functions as a proapoptotic molecule in PC3 and HeLa cells. Recently, a cancer-promoting role through activation of the ERK and AKT1 pathways has been described for miR-133b in other cervical cancer models [22]. To determine whether these signaling pathways are activated in HeLA or PC3 cells we performed Western blot analysis of miR-133b-transfected cells (Figure S5). In contrast to other cervical cell lines, miR-133b expression did not influence AKT1 or ERK phosphorylation in HeLa cells. As a caveat we should note that ERK phosphorylation was barely detectable. Nevertheless, it did not seem to be induced. This points to an ambivalent function of miR-133b, which can be pro- or antiapoptotic depending on cell type.

## Discussion

Herein, we used human cervix and prostate carcinoma cells as a model system for DR-triggered apoptosis to interrogate whether miR-133b is capable of modifying the cellular death response to TNFα, Fas/CD95 ligand (FasL) or TRAIL. Our findings reveal that miR-133b is a potent catalyzer of DR-mediated cell death. The accentuated apoptotic response of miR-133b transfectants is a direct consequence of synergistic alterations of their protein repertoire. Specifically, miR-133b directly impairs the expression of important antiapoptotic genes. We provide evidence for FAIM as a target of miR-133b. Originally discovered during a screen aiming to identify factors responsible for Fas/CD95 resistance in primary splenic B cells, FAIM has emerged as a highly conserved atypical regulator of DR-mediated apoptosis [32]. Since FAIM does not exhibit a significant primary sequence homology to any other known effector protein motif, its primary structure does not reveal any insights on how it can exert its antiapoptotic function.

At present, two major isoforms of FAIM have been described. The long isoform (FAIM-L) is present almost exclusively in neurons, whereas the short one (FAIM-S) displays a much broader expression pattern [40]. Over-expression studies in neurons revealed a protective role of FAIM-L against TNFα- and FasL-mediated apoptosis upstream of both caspase 8 and 3, and the ability of FAIM-S to promote neurite outgrowth by a mechanism involving activation of NF-κB [40], [41]. Noticeably, FAIM-L and -S share the same 3′-UTR and miR-133b binding site, implying that both are subjected to post-transcriptional regulation by miR-133b. In fact, enriched presence of miR-133b was identified in the midbrain region, where it regulates the maturation and function of dopaminergic neurons [42]. Based on this observation and the data provided here, it would be attractive to investigate the interplay between FAIM and miR-133b during DR-triggered apoptosis and neuron development in the midbrain.

Importantly, FAIM positively influences the expression of central antiapoptotic cellular FLICE-like inhibitory protein (cFLIP) in lymphocytes and hepatocytes *in-vivo*. Reduced levels of cFLIP would allow a better physical interaction of procaspase 8 with Fas/CD95 thus leading to more pronounced caspase activation and probably enhanced apoptosis [43]. To test whether this was also the case in miR-133b-transfected cells, cFLIP expression was analyzed. No significant difference of cFLIP between ctrl miR- and miR-133b-transfected cells could be observed at the mRNA or protein level (data not shown). A possible explanation for this could be that generation of a knockout mouse leads to complete absence of the deleted gene, whereas transient miR treatment merely results in a partial reduction (∼70%) of protein expression. Therefore, miR-133b-transfected cells might still contain sufficient FAIM to assure expression of cFLIP. As the first reported post-transcriptional regulator of FAIM and considering the high degree of conservation of miR-133b and FAIM across different species, the role of miR-133b during further biological processes should be a matter of future studies.

In addition to FAIM, we could further pinpoint the expression of an important antiapoptotic enzyme as miR-133b-dependent by using global proteome quantification techniques. GSTP1 belongs to a family of enzymes responsible for detoxification and protection of cells from attack by reactive species. GSTP1 expression is highly elevated in many neoplastic tissues and has been implicated in resistance to apoptosis [34]. GSTP1 was reported to regulate TNFα-triggered signaling through interaction with TNF receptor-associated factor 2 (TRAF2). As a consequence of this interaction, activation of apoptosis signal-regulating kinase 1 (ASK1) is impaired, and TNFα-mediated apoptosis is strongly disturbed [44]. Recently, miR-133a, a cognate molecule of miR-133b, was reported to regulate the expression of GSTP1 in head and neck squamous cell carcinoma (HNSCC) cells [45]. MiR-133a and b differ only in one base pair at the 3′- end of the molecules (G→A). This position is furthest away from the seed region, which is essential for miR:target interaction. Hence, it is likely that miR133a and b perform similar if not identical cellular functions by regulating the expression of a common pool of target genes. In addition, miR-133a and the co-transcribed miR-1 were recently described to exhibit a reduced expression in prostate and bladder cancer in which miR-133a targets Transgelin 2 (TAGLN2), a gene with oncogenic properties that was strongly downregulated in our pSILAC dataset [46], [47], [48].

So far, miR-133b has been almost exclusively described in the context of miR signatures from tumor samples or cancer cell lines and its potential for diagnostics and prognosis. Previous reports demonstrate a significant downregulation of miR-133b in transformed tissue compared to healthy controls [13], [14], [16], [18], [21], [38]. One recent report ascribes tumor-promoting functions to miR-133b in *in-vitro* and *in-vivo* models of cervical cancers [22]. This work focused on cervical cell lines other than HeLa cells, which were inspected for their expression levels of miR-133b. In this cell line miR-133b levels werefound to be slightly elevated compared to other cervical cancer cell lines. Our HeLa experiments point to a proapoptotic and presumably antitumorigenic role of miR-133b. Therefore it is conceivable that miR-133b fulfills different roles in HeLa cells and other cervical cancer cell lines. It is well known that the same molecule can have opposing roles in different cellular settings [49], [50]. Note, that differential results were obtained while examining the expression of miR-133b in cervical cancer compared to healthy tissue. One study reports upregulation of this miR as revealed by qRT-PCR whereas a sequencing approach and microarray analysis point to a repression of miR-133b in tumor tissue [22], [51], [52]. Further experiments will be necessary to clarify this conundrum of pro- or antiapoptotic functions of miR-133b in cervical and other types of cancer.

Herein, we addressed the question whether miR-133b is also downregulated in prostate cancer. We show that miR-133b expression is reduced in the majority of prostate cancers when compared to normal adjacent tissue. Remarkably, patients with a low abundance of miR-133b tend to experience biochemical relapse more frequently. Accordingly, transfection of a prostate tumor cell line with synthetic miR-133b mimics resulted in sequence-specific impairment of proliferation capacity, suggesting a functional relevance of the reduced miR-133b expression in cancerous prostate cells. Ongoing work focuses on elucidating the exact molecular mechanisms responsible for this phenotype.

Finally, our results identify miR-133b as a highly versatile and potent proapoptotic molecule with tumor suppressor properties. The evidence provided here, in combination with previous findings showing that miR-133b is concordantly repressed in various tumor types and, that it is capable of regulating the intrinsic apoptotic pathway and expression of important oncogenes, strongly suggests that downregulation of miR-133b represents an important step during tissue transformation [38]. Hence, further studies should aim at exploring the potential of miR-133b as molecular target for cancer therapy.

## Materials and Methods

### Ethics statement

The study was approved by the ethical board of the Charité University Hospital (EA1/153/07) and written informed consent has been obtained.

### Cell lines

HeLa and PC3 cells were obtained from the German Collection of Microorganisms and Cell Cultures (DSMZ) (Braunschweig, Germany). All cell lines were kept in culture under conditions recommended by the American Type Culture Collection (ATCC) (Manassas, VA, USA).

### Patients and tissue samples

Tumor tissue and normal adjacent tissue from 69 patients with prostate carcinoma were collected after radical prostatectomy at Charité University Hospital between 2001 and 2005. Samples were snap-frozen directly after surgery. Tumor areas were identified by haematoxylin and eosin staining and tumor and normal adjacent tissue was punch-biopsied with a 1-mm tissue microarray needle. Tumor content of the punches was histologically reevaluated to confirm a tumor content >90% in each sample. Frozen matched malignant and nonmalignant samples were collected in RNAlater stabilization reagent (Qiagen GmbH, Hilden, Germany). For all patients, the following clinicopathological information was available: Tumor classification according to the International Union Against Cancer (UICC) 2002 TNM system, tumor grading according to Gleason, follow-up time after surgery and follow-up prostate-specific antigen (PSA) values. Biochemical relapse was defined as the first PSA value after radical prostatectomy >0.1 and was confirmed by a subsequently elevated value. Additionally, only patients whose PSA levels dropped below detection limit after surgery were considered for analysis.

### Reagents, cytokines and death receptor ligands

For stimulation and apoptosis induction experiments recombinant human TNFα from Active Bioscience (Hamburg, Germany) was used; human activating antiFas/CD95 (Clone CH11) (αFas/CD95) was from Upstate (Temecula, CA, USA); soluble human recombinant TRAIL was purchased from Alexis Biochemicals (Farmingdale, NY, USA). For apoptosis inhibition the general caspase inhibitor z-Val-Ala-DL-Asp(Ome)-fluoromethylketone (Z-VAD-FMK) from BD-Biosciences (San Jose, CA, USA) was used.

### miR mimics and inhibitor molecules

For transfection experiments synthetic miR-133b (Catalog number PM10029), antimiR-133b (αmiR-133b; catalog number PM10029) and their respective scrambled controls: control miR (ctrl miR; catalog number AM17110) and control antimiR (ctrl αmiR; catalog number AM17010) were purchased from Ambion (Austin, TX, USA). Unless otherwise specified, negative control and miRs were used at 10-nM concentration. For inhibition experiments αmiRs or scrambled control were added at 30-nM concentration. All transfections were carried out using Lipofectamine 2000 from Invitrogen (Carlsbad, CA, USA) according to the manufacturer's protocol.

### Apoptosis assays

The day before transfection, HeLa or PC3 cells were seeded in 24-well plates at a density of 5×10^4^ cells/well. MiR-133b or ctrl miR was transfected either alone or together with ctrl or specific αmiR-133b. Forty-eight h after transfection, cells were stimulated for 6 h with the respective proapoptotic stimuli. Apoptotic cells were quantified by flow cytometry by measuring caspase activation status with caspase-specific FLICA™ apoptosis detection kits from Immunochemistry Technologies (Bloomington, MN, USA) as per manufacturer's instructions. 7-Amino-actinomycin D (7-AAD) was used to exclude cells with damaged cellular membrane from the caspase activation quantification. Propidium iodide (PI) incorporation was used for determining overall cell vitality. For this, treated cells were labeled with PI (Immunochemistry Technologies) and analyzed by flow cytometry.

### 3-(4,5-Dimethylthiazol-2-yl)-2,5 diphenyltetrazolium bromide (MTT) cell survival and proliferation assay

MTT was diluted in PBS to a final concentration of 5 mg/ml and sterile filtered. Before use, MTT stock solution was further diluted with cell culture medium (without FCS) to a final concentration of 100 µg/ml. To perform the assay, 50 µl of this solution were added to the culture plate containing transfected cells in 50 µl supernatant. Treated cells were incubated for another 5 h at 37°C and controlled hourly by light microscopy for formation and precipitation of violet formazan crystals. Next, cell culture supernatants were removed carefully, without disturbing the cell monolayer. Adherent cells were lysed with 100 µl 100% DMSO. Finally, DMSO samples containing diluted formazan crystals were transferred to ELISA plates and OD_570_ values were determined using a Spectramax 190 reader from Molecular Devices (Sunnyvale, CA, USA).

### Western blotting

For protein detection, PC3 or HeLa cells were transfected and incubated for 48 h. Protein lysates were generated by collecting and pooling the supernatants and adherent cells and washing them briefly with phosphate-buffered saline. Cell pellets were lysed by adding 1× Laemmli sample buffer containing 62.5 mM Tris-HCl, pH 6.8, 2% SDS, 25% glycerol, 0.01% bromophenol blue and 2-mercaptoethanol. Protein expression was analyzed by standard procedures for Western blotting. A list of antibodies used in this study can be found in Table S1. Proteins of interest were normalized to GAPDH and analyzed densitometrically using LAS-3000 CCD imaging system from Fujifilm (Tokyo, Japan) and AIDA Biopackage-2D (Raytest, Straubenhardt, Germany).

### RNA isolation and quantitative PCR

Total RNA for quantitative PCR was isolated with TRIzol from Invitrogen according to total RNA-isolation protocol recommended by the manufacturer and using glycogen (Ambion) as a carrier. Quantitative PCR of miR-133b and miR-130 was performed with TaqMan microRNA assays from Applied Biosystems using 100 ng total RNA for the reverse transcription step. For analysis of the protein-coding gene expression, 2 µg RNA were reverse transcribed using random hexamers (Fermentas, Burlington, Canada) and SuperScript III First-Strand Synthesis System for RT-PCR (Invitrogen). After reverse transcription all samples were subjected to DNAseI (Invitrogen) treatment in order to remove contaminating genomic DNA. All samples were analyzed with a 7900HT fast real-time PCR system (Applied Biosystems) and subjected to comparative ΔΔCt method by using human acidic ribosomal protein (HuPO) as the internal standard. For further information about all primers see Table S2.

### RNA isolation and quantitative PCR from primary prostate cancer tissue

Total RNA was extracted with the miRNeasy Mini Kit (Qiagen) according to manufacturer's recommendations. Quality and yield was monitored using a NanoDrop ND-100 photometer (Thermo Fisher Scientific Inc., Waltham, MA, USA) and a 2100 Bioanalyzer (Agilent Technologies, Santa Clara, CA, USA). Only samples with RNA Integrity Numbers (RIN) above 6 were used for subsequent analysis. Relative expression of miR-133b in prostate tissue was measured as previously described [53]. cDNA was reverse transcribed from 6.7 ng total RNA. Expression of miR-133b was normalized to miR-130b [53]. Standard curves were generated for each miRNA to allow for suboptimal efficiencies and to calculate arbitrary concentrations.

### Microarray analysis

Microarray experiments were performed as dual-color hybridizations. To compensate for dye-specific effects, a dye-reversal color-swap was applied. RNA labeling was performed with the Quick Amp Labeling Kit (Agilent Technologies). In brief, mRNA was reverse transcribed and amplified using an oligo-dT-T7-promotor primer and resulting cRNA was labeled with dyes. 1.25 µg of each labeled cRNA was fragmented and subsequently hybridized to whole human genome 44 k microarrays (AMADID-014850) according to the supplier's protocol (Agilent Technologies). Data files were further analyzed with the Rosetta Resolver Biosoftware, Build 7.2 (Rosetta Biosoftware, Seattle, WA, USA). A 1.5-fold change expression cut-off for ratio experiments was applied together with anticorrelation of ratio profiles rendering the microarray analysis highly significant (P-value>0.01), robust and reproducible. The data presented in this publication have been deposited in NCBI's Gene Expression Omnibus (GEO, http://www.ncbi.nlm.nih.gov/geo/) and are accessible through GEO Series accession number GSE24613.

### Pulsed stable isotope labeling with amino acids in cell culture (pSILAC)

Changes in the proteome of miR-transfected HeLa cells were analyzed by performing a slightly modified version of the pSILAC method described by Selbach *et. Al* [33]. Briefly, 1.5×10^6^ HeLa cells cultivated in normal light medium were transfected for 5 h with ctrl miR or miR-133b. Treated cells were next transferred to culture medium containing medium-heavy (M; ^13^C_6_-arginine and ^2^H_4_-lysine) or heavy (H; ^13^C_6_^15^N_4_-arginine and ^13^C_6_^15^N_2_-lysine) isotope-labeled amino acids and pulse labeled for another 40 h. All media were prepared using SILAC™ D-MEM and amino acids from Invitrogen and Thermo Fisher Scientific (Perbio Science). Finally, both groups of cells were stimulated in the same media for 6 h with 25 ng/ml TNFα and processed for downstream mass spectrometry analysis. Briefly, three replicates of pSILAC-labeled protein lysates were separated by SDS-PAGE, the Coomassie G-250 gel lanes were subsequently in-gel digested with trypsin, followed by analysis of the tryptic peptides using a nanoLC with an 87-min gradient of increasing acetonitrile from 7 to 40% which was directly coupled to an LTQ-Orbitrap XL mass spectrometer operated in data-dependent mode and lock mass option. Raw data were analyzed using MaxQuant [54], the human IPI database, and Mascot 2.2. See File S1 for a detailed description.

### 3′- untranslated region (3′-UTR) cloning, mutation and luciferase reporter assay

Validation of miR-targets was performed by cloning complete 3′-UTR of predicted genes. For this, PCR products designed to have restriction sites for *XhoI* and *NotI* (for primer sequence please refer to Table S3) were subcloned into pCR2.1-TOPO (Invitrogen) and subsequently cloned into the psiCHECK-2 plasmid from Promega (Madison, WI, USA). Putative miRNA binding sites were mutated using the QuikChange Site-Directed Muta-genesis Kit (Stratagene). Primer sequences designed to generate the mutations are displayed in Table S3. Insert's correctness of all constructs was confirmed by sequencing. Luciferase reporter assays were performed by cotransfecting HeLa cells with psiCHECK-2 constructs containing wildtype or mutated 3′-UTRs and ctrl miR or miR-133b. Forty-eight h post-transfection, cells were rinsed once with PBS and lysed with passive lysis buffer (Promega). Luciferase activity was measured in a Victor Luminometer (Perkin Elmer, Waltham, MA, USA) using the Dual-Luciferase Reporter Assay System from Promega. Total *Renilla* luciferase activity was calculated by normalizing to firefly luciferase in order to correct for differences in transfection efficiency.

### Statistics

Unless otherwise specified, data shown are representative of at least three independent experiments. Statistical significance was calculated by two-tailed Student's *t*-test and p<0.05 was considered significant. Statistical analyses of RT-qPCR data from primary tissue was performed using PASW statistics 18.0.0 (SPSS Inc., Chicago, IL, USA), GraphPad version 5.00 (GraphPad Software Inc. La Jolla, CA, USA) and Medcalc version 11.0 (MedCalc Software, Mariakerke, Belgium). Kolmogorov-Smirnov, Wilcoxon signed rank test and Spearman correlation were used. All tests were performed two-tailed and p<0.05 was considered significant. Receiver operating characteristic (ROC) curves were calculated and univariate logistic regression was performed to determine the discriminative power. For survival analyses Kaplan-Meier approach (log rank test) and Cox proportional hazard regression were used.

## Supporting Information

Figure S1 **Kaplan–Meier plot of miR-133b.** Ratio of miR-133b in prostate tumor tissue versus normal adjacent tissue was dichotomized according to the median expression ratio. Differences in recurrence-free survival were analyzed by log rank test.
(PDF)

Figure S2 **Ability of miR-133b to discriminate prostate cancer tissue and normal adjacent tissue.** ROC analysis was performed on miR-133b expression. Dotted line indicates an AUC of 0.5.
(PDF)

Figure S3 **miR-133b controls expression of GSTP1 in PC3 cells.** Western blot and densitometric analysis of GSTP1 expression. PC3 cells were transfected with miR-133b alone or together with ctrl αmiR or a specific αmiR-133b. After 48 h, cellular protein lysates were prepared and GSTP1 expression was assessed by Western blot. GAPDH was used as an internal loading standard. Ctrl miR-transfected cells were used as a reference for densitometric quantification of protein band intensity.
(PDF)

Figure S4 **miR-133b controls expression of FAIM in PC3 cells.** Western blot and densitometric analysis of FAIM expression. PC3 cells were transfected with miR-133b alone or together with ctrl αmiR or a specific αmiR-133b. After 48 h, cellular protein lysates were prepared and FAIM expression was assessed by Western blot. β-actin was used as an internal loading standard. Ctrl miR-transfected cells were used as a reference for densitometric quantification of protein band intensity.
(PDF)

Figure S5 **Western blots of ERK and AKT1 and their phosphorylated modifications to determine activation status of these pathways after miR-133b transfection.** HeLa and PC3 cells were transfected with miR-133b or a control miR (cntrl miR). 48 hours posttransfection whole cell extracts were prepared and the expression of A) AKT1/p-AKT1 and B) ERK/p-ERK was assessed using specific antibodies (AKT1, C67E7; p-AKT, D9E; ERK, 137F5; p-ERK, Y204, all antibodies available at Cell Signaling, Danvers, MA, USA). Since there is a big difference in the molecular weight of GAPDH and AKT1/p-AKT1 they could be probed on the same blot whereas for ERK/p-ERK and GAPDH different blots had to be used.
(PDF)

Table S1 **Antibodies used for Western blot analysis.**
(PDF)

Table S2 **Primer sequences used for PCR.**
(PDF)

Table S3 **Primers used for generation of luciferase reporter constructs**
(PDF)

File S1 **Detailed description of the pSILAC protocol.**
(PDF)
