# Supplementary figures and images for: MiR-133b Targets Antiapoptotic Genes and Enhances Death Receptor-Induced Apoptosis
Source: PLoS One. 2012 Apr 20;7(4):e35345. doi: 10.1371/journal.pone.0035345 (PMC3332114; doi:10.1371/journal.pone.0035345)

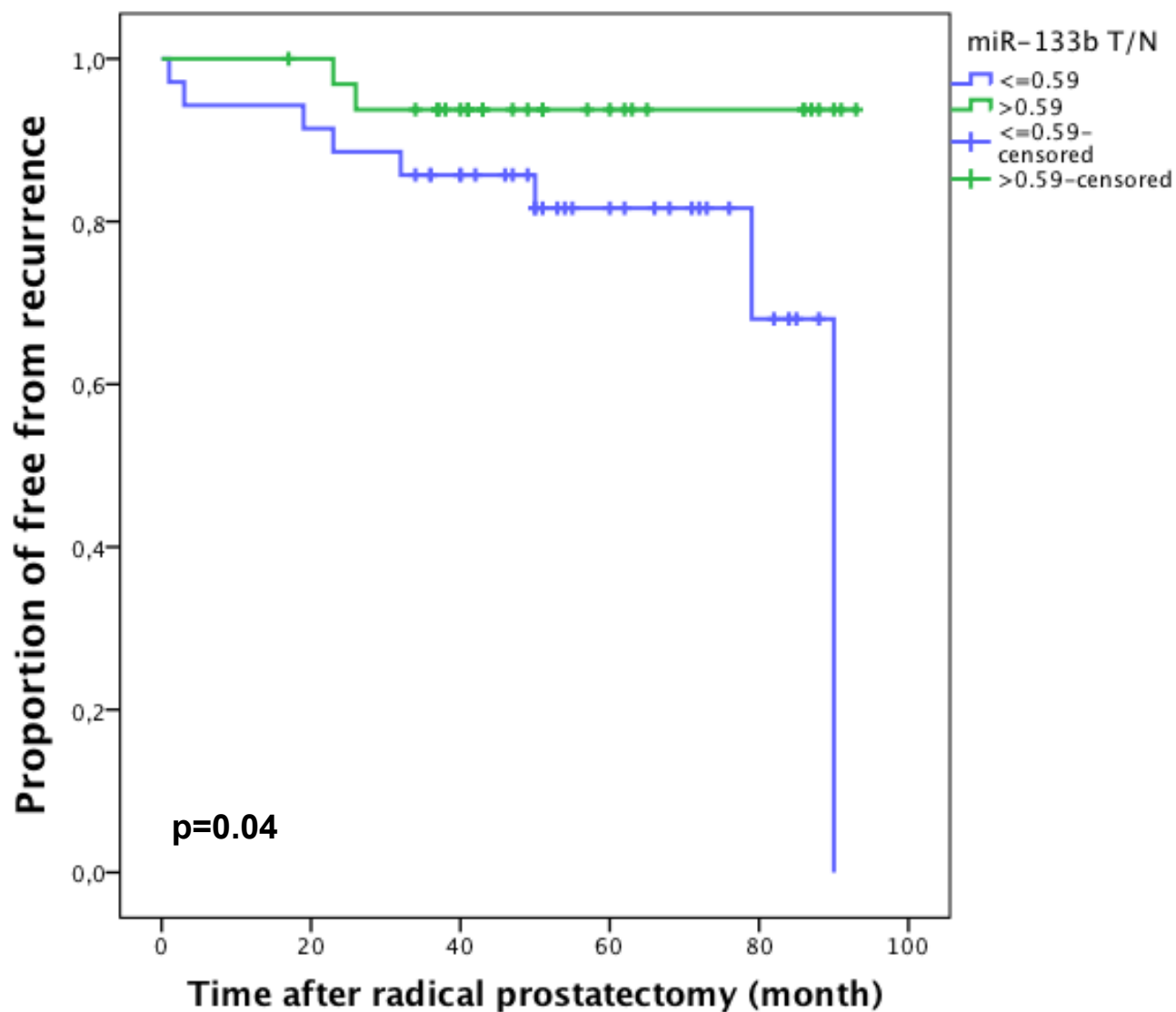

Supplement: Figure S1 — Kaplan–Meier plot of miR-133b. Ratio of miR-133b in prostate tumor tissue versus normal adjacent tissue was dichotomized according to the median expression ratio. Differences in recurrence-free survival were analyzed by log rank test. (PDF) [file pone.0035345.s001.pdf]

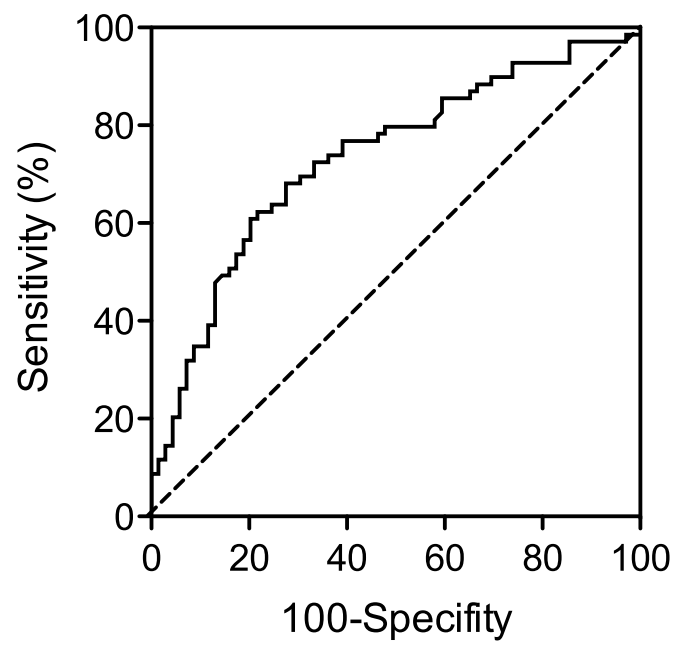

Supplement: Figure S2 — Ability of miR-133b to discriminate prostate cancer tissue and normal adjacent tissue. ROC analysis was performed on miR-133b expression. Dotted line indicates an AUC of 0.5. (PDF) [file pone.0035345.s002.pdf]

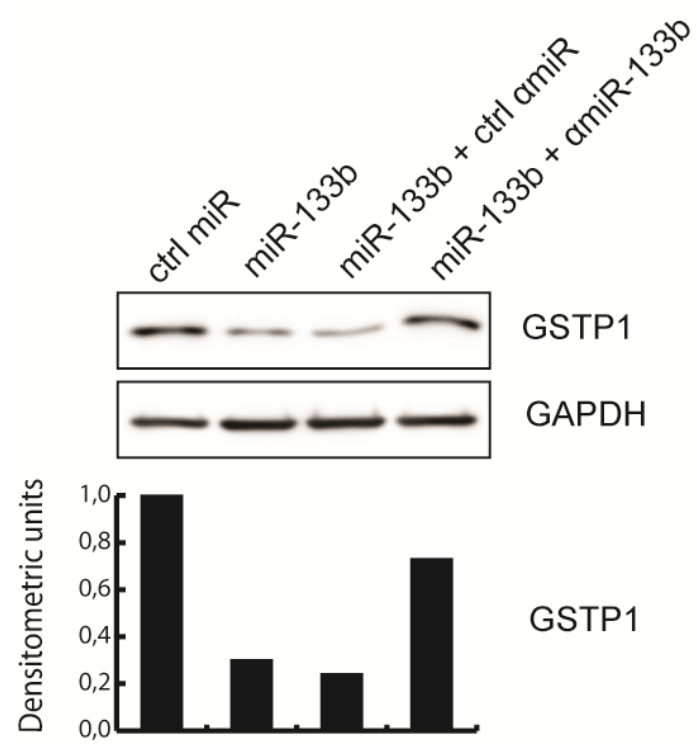

Supplement: Figure S3 — miR-133b controls expression of GSTP1 in PC3 cells. Western blot and densitometric analysis of GSTP1 expression. PC3 cells were transfected with miR-133b alone or together with ctrl αmiR or a specific αmiR-133b. After 48 h, cellular protein lysates were prepared and GSTP1 expression was assessed by Western blot. GAPDH was used as an internal loading standard. Ctrl miR-transfected cells were used as a reference for densitometric quantification of protein band intensity. (PDF) [file pone.0035345.s003.pdf]

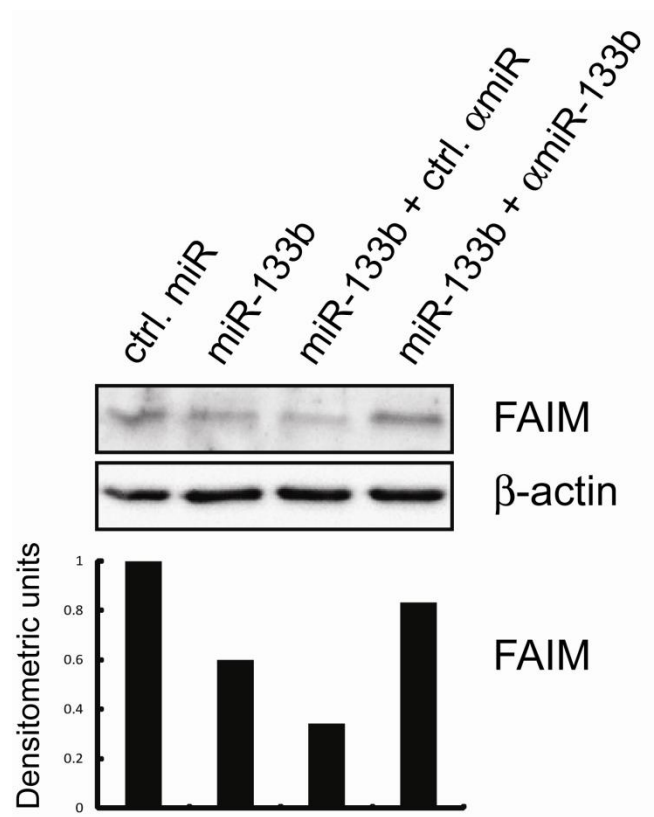

Supplement: Figure S4 — miR-133b controls expression of FAIM in PC3 cells. Western blot and densitometric analysis of FAIM expression. PC3 cells were transfected with miR-133b alone or together with ctrl αmiR or a specific αmiR-133b. After 48 h, cellular protein lysates were prepared and FAIM expression was assessed by Western blot. β-actin was used as an internal loading standard. Ctrl miR-transfected cells were used as a reference for densitometric quantification of protein band intensity. (PDF) [file pone.0035345.s004.pdf]

**A**

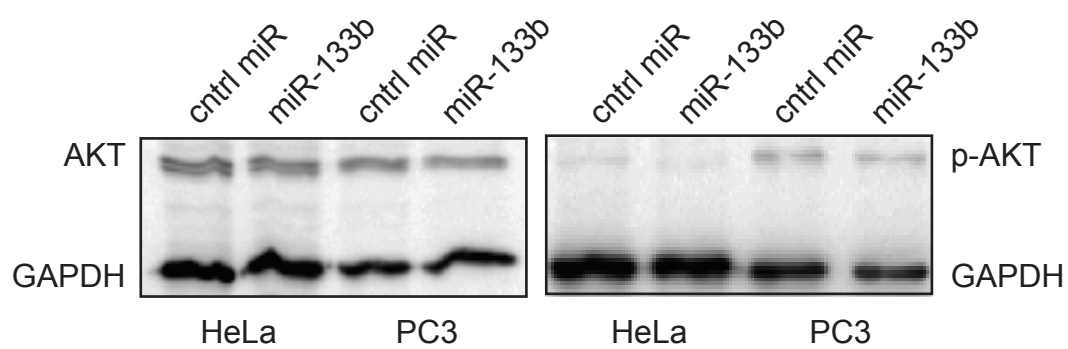

**B**

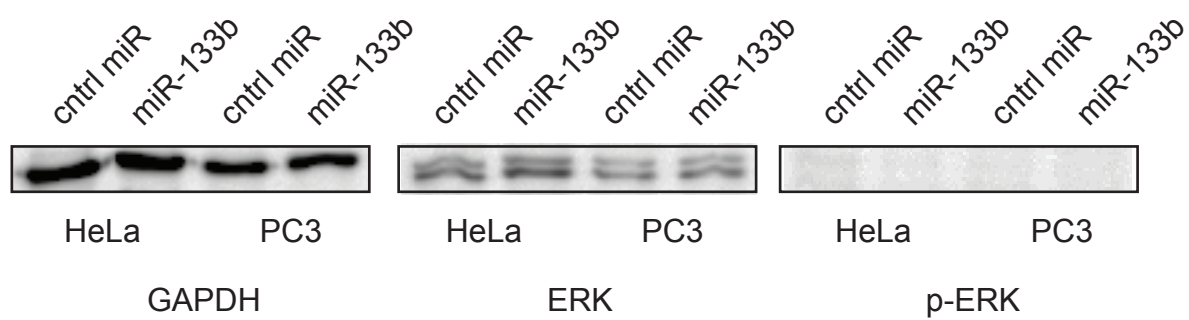

Supplement: Figure S5 — Western blots of ERK and AKT1 and their phosphorylated modifications to determine activation status of these pathways after miR-133b transfection. HeLa and PC3 cells were transfected with miR-133b or a control miR (cntrl miR). 48 hours posttransfection whole cell extracts were prepared and the expression of A) AKT1/p-AKT1 and B) ERK/p-ERK was assessed using specific antibodies (AKT1, C67E7; p-AKT, D9E; ERK, 137F5; p-ERK, Y204, all antibodies available at Cell Signaling, Danvers, MA, USA). Since there is a big difference in the molecular weight of GAPDH and AKT1/p-AKT1 they could be probed on the same blot whereas for ERK/p-ERK and GAPDH different blots had to be used. (PDF) [file pone.0035345.s005.pdf]
