# Supplementary material for: MiR-133b Targets Antiapoptotic Genes and Enhances Death Receptor-Induced Apoptosis
Source: PLoS One. 2012 Apr 20;7(4):e35345. doi: 10.1371/journal.pone.0035345 (PMC3332114; doi:10.1371/journal.pone.0035345)
Supplement: Table S1 — Antibodies used for Western blot analysis. (PDF) [file pone.0035345.s006.pdf]

Supplementary Table 1. Antibodies used for Western blot analysis of protein expression

Specificity	Host species	Clone	Dilution	Supplier
Anti-mouse IgG HRP-linked	Goat	Polyclonal	1:3000	Cell Signaling (Danvers, MA, USA)
Anti-rabbit IgG HRP-linked	Goat	Polyclonal	1:3000	Cell Signaling (Danvers, MA, USA)
FAIM-s	Rabbit	Polyclonal	1:5000	AntibodyBcn (Barcelona, Spain)
GAPDH	Rabbit	14C10	1:1000	Cell Signaling (Danvers, MA, USA)
GSTP1	Mouse	3F2	1:1000	Cell Signaling (Danvers, MA, USA)
PARP-1	Rabbit	Polyclonal	1:1000	Cell Signaling (Danvers, MA, USA)
