# Supplementary material for: MiR-133b Targets Antiapoptotic Genes and Enhances Death Receptor-Induced Apoptosis
Source: PLoS One. 2012 Apr 20;7(4):e35345. doi: 10.1371/journal.pone.0035345 (PMC3332114; doi:10.1371/journal.pone.0035345)
Supplement: Table S2 — Primer sequences used for PCR. (PDF) [file pone.0035345.s007.pdf]

**Supplementary Table 2**. Primer sequences used for PCR

| Primer name | Primer sequence 5´→ 3´ |
|---|---|
| HuPO_fw | GCTTCCTGGAGGGTGTCC |
| HuPO_re | GGACTCGTTTGTACCCGTTG |
| GSTP1-Mix | Quantitect (Qiagen) QT00086401 |
| FAIM-Mix | Quantitect (Qiagen) QT01022147 |
