# Supplementary material for: MiR-133b Targets Antiapoptotic Genes and Enhances Death Receptor-Induced Apoptosis
Source: PLoS One. 2012 Apr 20;7(4):e35345. doi: 10.1371/journal.pone.0035345 (PMC3332114; doi:10.1371/journal.pone.0035345)
Supplement: Table S3 — Primers used for generation of luciferase reporter constructs (PDF) [file pone.0035345.s008.pdf]

Supplementary Table 3. Primers used for generation of 3´-UTR luciferase reporter constructs

Primer name	Primer sequence 5´→ 3´
FAIM-3´-UTR _fw	**ctcgag**[1] TGAATTTTCATCTTAAGAAGTAAAGATCAG
FAIM-3´-UTR _re	**gcggccgc**[2] AAAAGTATTTATTATAGTAAAGGTTACTGTTGT
GSTP1-3´-UTR _fw	**ctcgag** AGTGAGGGTTGGGGGGA
GSTP1-3´-UTR _re	**gcggccgc** AGCTCTCTTAGAAATTTTATTGGTCCT
FAIMmut-5	GACAAATGGCAAATAAGATATCTGACTAATGTTTTACAACAGTAAC
FAIMmut-3	GTTACTGTTGTAAAACATTAGTGACATATCTTATTTGCCATTTGTC
GSTP1mut-5	GTTGCCTTCCTTTCTCCATAAAATTTCTAAGAGAGCT
GSTP1mut-3	AGCTCTCTTAGAAATTTTATGGAGAAAGGAAGGCAAC

[1] XhoI restriction sequence
[2] NotI restriction sequence
