# Supplementary material for: MiR-133b Targets Antiapoptotic Genes and Enhances Death Receptor-Induced Apoptosis
Source: PLoS One. 2012 Apr 20;7(4):e35345. doi: 10.1371/journal.pone.0035345 (PMC3332114; doi:10.1371/journal.pone.0035345)
Supplement: File S1 — Detailed description of the pSILAC protocol. (PDF) [file pone.0035345.s009.pdf]

Supplementary Materials and Methods

Down-stream processing of pSILAC samples

1) Protein lysate generation and SDS-PAGE analysis

Cell pellets were resuspended in lysis buffer (25 mM Tris-HCl, 50 mM KCl, 3 mM EDTA, 1% Triton X-100, 5 mM β-mercaptoethanol, pH 7.1) supplemented with protease (3 mM benzamidine, 10 μM leupeptin, and 1 mM PMSF) and phosphatase inhibitors (30 mM NaF, 1 mM Na_3VO_4, 20 mM $Na_4P_2O_7$) and homogenized using a pestle pellet. Finally, lysates were centrifuged for 10 min at 16,000 g and 4°C. SDS-PAGE was performed with 4% stacking gel and 10% separation gel using a Mini-Protean 3 cell (Bio-Rad, Oslo, Norway). Gels were stained with Coomassie Brilliant Blue G-250 (Serva, Heidelberg, Germany). Fixation was performed with 50% ethanol/2% phosphoric acid for 1 h, incubation with 34% ethanol/2% phosphoric acid/17% ammonium sulfate for 1 h, and staining with 20% methanol/10% phosphoric acid/10% ammonium sulfate for 1 h. Finally, the gels were washed once for 30 min with 25% ethanol and three times with water.

2) Nano-LC/LTQ-Orbitrap mass spectrometry

The Coomassie G-250 stained single gel lanes of three biological replicates were excised for in-gel digestion with 0.1 μg of trypsin (Promega, Madison, WI, USA) in 20 μL 25 mM ammonium bicarbonate, pH 7.8 at 37°C for 16 h. The dried peptides were dissolved in 10 μL 1% formic acid in water and 5 μl were injected into an Ultimate 3000 nano-LC system (Dionex, Sunnyvale CA, USA) connected to a linear quadrupole ion trap-orbitrap (LTQ-Orbitrap XL) mass spectrometer

(ThermoScientific, Bremen, Germany) equipped with a nanoelectrospray ion source. An Acclaim PepMap 100 column (C18, 3 µm, 100 Å) (Dionex) with a capillary of 12 cm bed length was used for separation by liquid chromatography. A flow rate of 300 nL/min was employed with a solvent gradient of 7% B to 40% B in 87 min, then 40% to 80% B in 8 min and subsequently from 40% to 80% B in 8 min. Solvent A was 0.1% formic acid, whereas aqueous 90% acetonitrile in 0.1% formic acid was used as solvent B.

The mass spectrometer was operated in the data-dependent mode to automatically switch between Orbitrap-MS and LTQ-MS/MS acquisition. Survey full scan MS spectra (from m/z 300 to 2,000) were acquired in the Orbitrap with resolution R = 60,000 at m/z 400 (after accumulation to a target of 1,000,000 charges in the LTQ). The method used allowed sequential isolation of the most intense ions, up to six, depending on signal intensity, for fragmentation on the linear ion trap using collisional-induced dissociation (CID) at a target value of 100,000 charges. For accurate mass measurements the lock mass option was enabled in MS mode and the polydimethylcyclosiloxane (PCM) ions generated in the electrospray process from ambient air were used for internal recalibration during the analysis (1). Target ions already selected for MS/MS were dynamically excluded for 90 s. Other instrument parameters have been previously described (2).

3) Data analysis

Instrument raw data was processed and quantified using MaxQuant (3). For processing, the top 6 MS/MS peaks per 100 Da were used to generate msn-files. These msn-files were searched against the human IPI database (v. 3.62, 83685 sequences) containing both reversed sequences and contaminants using an in-

house version of the Mascot search engine (v.2.2.1). A mass tolerance of 0.5 Da was used for MS/MS fragments. Trypsin was used as protease allowing up to one missed cleavage and peptide charge 2^+ and 3^+. As variable modifications oxidation (met) and N-acetyl (protein) was allowed. At least two peptides, and one of them being a unique peptide, was required for protein identification as well as an FDR of 1% were applied. For protein quantification, at least two ratio counts were required and razor peptides were included in the calculation of protein ratios. To assess the regulated proteins, the Grubbs test for outliers was applied. First the distribution of protein ratios were iteratively checked for outliers using the Grubbs test, and every outlier removed. The remaining population mean and standard deviation (SD) (calculated in log space) were used to describe the un-regulated population. All proteins outside 1.96 SD of this population were considered to be regulated

1. Olsen JV, de Godoy LM, Li G, Macek B, Mortensen P, Pesch R , et al. Parts per million mass accuracy on an Orbitrap mass spectrometer via lock mass injection into a C-trap. Mol Cell Proteomics 2005 Dec; 4(12): 2010-2021.

2. Koehler CJ, Strozynski M, Kozielski F, Treumann A, Thiede B. Isobaric peptide termini labeling for MS/MS-based quantitative proteomics. J Proteome Res 2009 Sep; 8(9): 4333-4341.

3. Cox J, Mann M. MaxQuant enables high peptide identification rates, individualized p.p.b.-range mass accuracies and proteome-wide protein quantification. Nat Biotechnol 2008 Dec; 26(12): 1367-1372.
